# High-dose tranexamic acid reduces blood loss in postpartum haemorrhage

**DOI:** 10.1186/cc10143

**Published:** 2011-04-15

**Authors:** Anne-Sophie Ducloy-Bouthors, Brigitte Jude, Alain Duhamel, Françoise Broisin, Cyril Huissoud, Hawa Keita-Meyer, Laurent Mandelbrot, Nadia Tillouche, Sylvie Fontaine, Françoise Le Goueff, Sandrine Depret-Mosser, Benoit Vallet, Sophie Susen

**Affiliations:** 1 Pole d'Anesthésie-Réanimation, CHU Lille, 2 avenue Oscar Lambret, Lille F-59037, France; 2 Pole d'Hématologie Transfusion, CHU Lille, 2 avenue Oscar Lambret, Lille F-59037, France; 3 EA2693, Université Lille Nord de France, 1 place de Verdun, Lille F-59045, France; 4 Pole de Santé Publique, CHU Lille, 2 avenue Oscar Lambret, Lille F-59037, France; 5 EA2694, Université Lille Nord de France, 1 place de Verdun, Lille F-59045, France; 6 Pole d'Anesthésie-Réanimation, Hôpital de la Croix Rousse, Hôpitaux civils de Lyon, 93 grande rue de la Croix-Rousse, Lyon F-69000, France; 7 Pole d'obstétrique, Hôpital de la Croix Rousse, Hôpitaux civils de Lyon, 93 grande rue de la Croix-Rousse, Lyon F-69004, France; 8 Service d'Anesthésie-Réanimation, CHU Louis Mourier, Assistance Publique des Hôpitaux de Paris, 178 rue des Renouillers, Colombes F-92701, France; 9 Université Paris 7 - Diderot, 5 rue Thomas Mann, Paris F-75013, France; 10 Service d'obstétrique, CHU Louis Mourier, Assistance Publique des Hôpitaux de Paris, 178 rue des Renouillers, Colombes F-92701, France; 11 Service d'Anesthésie-Réanimation, Maternité Monaco, rue Desandrouins, centre hospitalier, Valenciennes F-59300, France; 12 Service d'Anesthésie-Réanimation, Maternité Paul Gellée, 91 avenue Julien Lagache, centre hospitalier, Roubaix F-59100, France; 13 Pole d'Obstétrique, CHU Lille, 2 avenue Oscar Lambret, Lille F-59037, France; 14 EA2689, Université Lille Nord de France, 1 place de Verdun, Lille F-59045, France; 15 Pole recherche, CHU Lille, 2 avenue Oscar Lambret, Lille F-59037, France

## Abstract

**Introduction:**

Our purpose in conducting this study was to determine whether administration of high-dose tranexamic acid (TA) at the time of diagnosis of postpartum haemorrhage (PPH) could reduce blood loss.

**Methods:**

This was a randomised, controlled, multicentred, open-label trial. Women with PPH >800 mL following vaginal delivery were randomly assigned to receive TA (loading dose 4 g over 1 hour, then infusion of 1 g/hour over 6 hours) or not. In both groups, packed red blood cells (PRBCs) and colloids could be used according to French guidelines. The use of additional procoagulant treatments was permitted only in cases involving intractable bleeding. The primary objective was to assess the efficacy of TA in the reduction of blood loss in women with PPH, and the secondary objectives were the effect of TA on PPH duration, anaemia, transfusion and the need for invasive procedures.

**Results:**

A total of 144 women fully completed the protocol (72 in each group). Blood loss between enrolment and 6 hours later was significantly lower in the TA group than in the control group (median, 173 mL; first to third quartiles, 59 to 377) than in controls (221 mL; first to third quartiles 105 to 564) (*P *= 0.041). In the TA group, bleeding duration was shorter and progression to severe PPH and PRBC transfusion was less frequent than in controls (*P *< 0.03). Invasive procedures were performed in four women in the TA group and in seven controls (*P *= NS). PPH stopped after only uterotonics and PRBC transfusion in 93% of women in the TA group versus 79% of controls (*P *= 0.016). Mild, transient adverse manifestations occurred more often in the TA group than in the control group (*P *= 0.03).

**Conclusions:**

This study is the first to demonstrate that high-dose TA can reduce blood loss and maternal morbidity in women with PPH. Although the study was not adequately powered to address safety issues, the observed side effects were mild and transient. A larger international study is needed to investigate whether TA can decrease the need for invasive procedures and reduce maternal morbidity in women with PPH.

**Trial registration:**

Controlled Trials ISRCTN09968140.

## Introduction

Postpartum haemorrhage (PPH) remains a leading cause of early maternal death, accounting for about 300,000 deaths worldwide every year, and of morbidity related to anaemia, blood transfusion and haemorrhage-related ischaemic complications [1,2]. PPH is poorly predictable, but its direct causes are mainly uterine atony, trauma to the genital tract and retained placenta [3-5]. Accordingly, detailed guidelines have been issued for optimal use of obstetric interventions and uterotonic drugs [6]. In contrast, haemostatic abnormalities in this setting have long been considered consequences of uncontrolled bleeding, not deserving of early specific treatment. Thus, haemostatic drugs are not routinely used as a first-line intervention in PPH [6,7].

This concept was recently challenged by the demonstration of a relationship between fibrinogen decrease and outcome [8]. At the same time, it was recognized that extensive tissue injury can shift the haemostatic equilibrium toward increased fibrinolysis, contributing to coagulopathy and bleeding [9]. Antifibrinolytic agents, mainly tranexamic acid (TA) and aprotinin, have been demonstrated to reduce blood loss and transfusion requirements in various elective surgeries [10]. Moreover, the Clinical Randomisation of an Antifibrinolytic in Significant Haemorrhage (CRASH-2) study demonstrated that TA safely reduces the risk of death in bleeding trauma patients [11]. In the field of obstetrics, three randomised, controlled trials [12-14] have suggested that TA administration in women after vaginal or elective caesarean delivery reduces blood loss and the incidence of PPH, with a pooled relative risk for PPH of 0.44 (95% confidence interval, 0.31 to 0.64) [15]. However, such a strategy implies that the drug must be administered to every woman, an option that needs careful evaluation in terms of the benefit-risk ratio before it is widely implemented. A more efficient approach could be to administer TA after the onset of PPH, as recently suggested [16]. However, no study has yet assessed the efficacy and risk of such a strategy.

Therefore, we designed a prospective, multicentred, randomised, controlled study to analyze the effects of TA administered intravenously at the time PPH is diagnosed. The primary objective of the study was to assess the efficacy of TA in the reduction of blood loss in PPH, while secondary objectives were to assess the effect of TA on (1) duration of bleeding; (2) anaemia; (3) need for invasive procedures such as hysterectomy, surgical artery ligatures and embolisation; and (4) need for transfusion.

## Materials and methods

### Trial framework

The trial was conducted between 2005 and 2008 in eight French obstetric centres (five tertiary care centres (102 patients) and three secondary care obstetric units (50 patients). The protocol was approved by the ethics committee of the University Hospital of Lille in June 2005 (CP05-07, CCPP nord-ouest 4, France), and data concealment was validated by the French Commission Informatique et Liberté (CNIL-MRO1). All pregnant women who received prenatal care in the participating centres were given information about this protocol during routine third-trimester visits. The women gave their written consent before entering the study in accordance with the Declaration of Helsinki. This study was funded and monitored by the French Ministry of Health (Programme Hospitalier National de Recherche Clinique, 2004 no. 1915). The funding source approved the study but had no role in the collection, analysis or interpretation of data; in the writing of the report; or in the decision to submit the paper for publication.

### Study design and patient eligibility criteria

**Figure 1:**
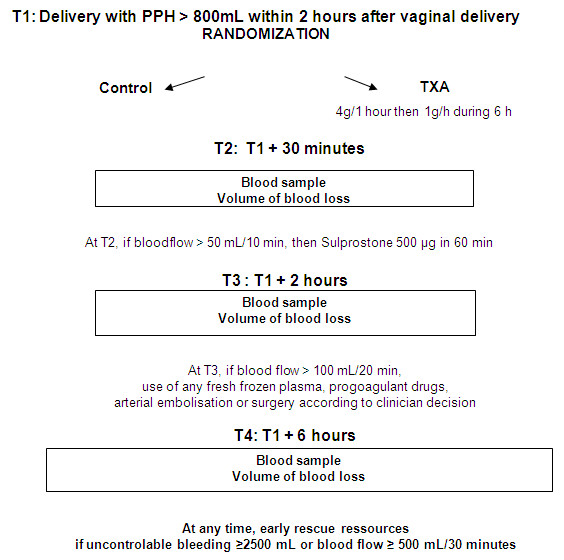
**Diagram showing the study design**. PPH, postpartum haemorrhage; TXA, tranexamic acid.

This academic multicentred, randomised, controlled, open-label study evaluated the efficacy and safety of TA in women with PPH. The design of the study is presented Figure 1. In each participating centre, an under-buttocks drape with a graduated collection pouch (Vygon, Ecouen, France) was placed immediately after each vaginal delivery to measure blood loss in the postpartum period. Overestimation of blood loss because of the addition of antiseptic or saline solutions used for washing or bladder catheterization was avoided. Midwives unaware of the group allocation measured the volume of haemorrhage in the graduated collection bag at each time point. Gauze was strictly kept for weighing. Baseline and final blood loss measurement were quantified and verified by weighing the pouch and the gauze. All patients with PPH >500 mL were managed according to the same timing according to French practice guidelines [16]: bladder catheter, manual removal of retained placenta, genital tract examination, uterine exploration and oxytocin (30 U/30 minutes), followed, and if these procedures were inefficacious, sulprostone was administered (500 μg in 1 hour) without any procoagulant treatment. Patients with PPH >800 mL were included in the study. Exclusion criteria were age <18 years, absence of informed consent, caesarean section, presence of known haemostatic abnormalities before pregnancy and history of thrombosis or epilepsy.

Immediately after inclusion, patients were randomised to receive either TA (TA group) or no antifibrinolytic treatment (control group). The randomisation sequence was generated by a centralized computer, and randomisation was balanced by centre. In the TA group, a dose of 4 g of TA was mixed with 50 mL of normal saline and administered intravenously over a 1-hour period. After the loading dose infusion, a maintenance infusion of 1 g/hour was initiated and maintained for 6 hours. This high dose was chosen as the best dose for the reduction of bleeding in high-risk cardiac surgery [17,18] and was administered to reduce significant active haemorrhage of more than 800 mL the clinical course of which might be life-threatening.

At four time points (T1 = inclusion, T2 = T1 + 30 minutes, T3 = T1 + 2 hours and T4 = T1 + 6 hours), the graduated collection pouch was replaced and the volume of blood loss was recorded. Blood haemoglobin level was also measured (HemoCue; HemoCue Meaux France). Blood samples were collected and tested for haemoglobin concentration and hematocrit. Bleeding was considered to have stopped when the flow was <50 mL/10 minutes.

In both study groups, packed red blood cells (PRBCs) and colloids could be used according to French guidelines. Vascular loading was as follows: crystalloid Ringer's lactate solution (Macoflex; Boulogne Billancourt, France) (500 mL) and the gelatin plasma expander Gelofusine 4% (B-Braun Medical, Boulogne Billancourt, France) (500 mL) for the first bleeding litre, then an infusion of gelatin was administered to compensate for blood loss (vol/vol). When blood loss exceeded 2,500 mL, loading was partially supported by an infusion of fresh frozen plasma (FFP). According to French guidelines, infusion of PRBCs was indicated when the patient's haemoglobin level was <8 g/dL.

In both study groups, the use of additional procoagulant treatment (FFP, platelets and fibrinogen concentrate) was not permitted before T3. However, at any time in both groups, additional procoagulant treatments or invasive procedures could be used in cases of intractable bleeding (PPH >2,500 mL or blood flow >500 mL/30 minutes).

According to national guidelines, postpartum thromboprophylaxis was carried out with low-molecular-weight heparin 50 IU/kg/day in the patients in severe condition in both groups from day 1 until the inflammatory syndrome disappeared.

### Criteria for evaluation

The primary end point was the volume of blood loss between T1 and T4. Secondary end points were duration of bleeding and the impact of TA on PPH-related outcome (decrease in haemoglobin concentration; transfusion of PRBCs at T4 and at day 42; and the need for invasive procedures (uterine artery embolisation or ligature, hysterectomy), late postpartum curettage or general outcome (intensive care unit stay, use of any vasopressors, dyspnoea, renal and multiple organ failure)). Severe PPH was defined by Charbit *et al. *[8] as exhibiting one of the following criteria: peripartum decrease of haemoglobin >4 g/dL, with the last haemoglobin value before delivery considered as the reference; transfusion of at least 4 U of PRBCs; invasive haemostatic intervention; or death. Evaluation of each end point was performed by investigators blinded to treatment allocation.

### Side effects

Although this study was not powered to address safety issues, side effects that could be related to TA were analyzed. Major side effects (thrombotic events, renal failure or seizures) and minor side effects were reported at each time point and at day 42. With respect to venous thrombosis, clinical signs of superficial or deep thrombosis were collected, and ultrasonography was performed as soon as the signs were detected.

### Sample size calculation

In a preliminary study, the mean ± standard deviation (SD) volume of PPH observed at T4 was 1,340 mL ± 490 mL. To demonstrate a decrease of 20% in the volume of PPH in the TA group, the number of patients had to be 144 for a type I error of 5% and a power of 90% [12].

### Statistical methods

Anonymous data were managed by an independent operator (Altizem, Nanterre, France) after double data acquisition. Results are expressed as means ± SD in cases of normal distribution and as medians and interquartile ranges otherwise. The normality of the distributions was tested using the Shapiro-Wilk test. Comparisons between groups were performed using the χ^2 ^test or Fisher's exact test for categorical variables. For numerical variables, we used Student's *t*-test in cases of normal distribution and the Mann-Whitney *U *test otherwise. All analyses involving the volumes of PPH were adjusted for the volume of blood loss between birth and T1 and for the centre. Since the distributions of the volumes of PPH were not normal, these parameters were analyzed using the nonparametric procedure recommended by Conover and Iman [19]. For the primary end point, comparison between the two groups was performed using covariance analysis. The time course of blood loss was studied using analysis of variance for repeated measurements. *Post hoc *analyses were performed using the Bonferroni correction. For the primary objective, analyses were performed both per protocol and on an intention-to-treat (ITT) basis. The duration of bleeding was analyzed by using the Kaplan-Meier method and compared across groups by using the logrank test. All statistical analyses were performed using SAS software (SAS Institute, Cary, NC, USA). A *P *value < 0.05 was considered statistically significant.

## Results

**Figure 2:**
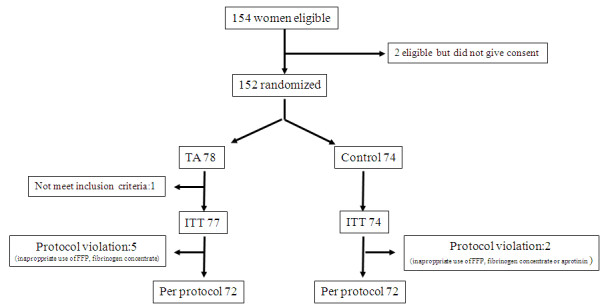
**Diagram showing the study profile**. ITT, intention to treat; FFP, fresh frozen plasma.

Among 154 women who were eligible for inclusion, 2 did not agree to be included, so 152 were included. Among them, one woman was later found not to meet the inclusion criteria and seven other women (*n *= 5 in the TA group and *n *= 2 in the control group) had protocol violations (inappropriate infusion of additional procoagulant treatments, such as FFP, fibrinogen concentrate, aprotinin or a large amount of PRBCs before T3 in the absence of intractable haemorrhage). Therefore, 144 women fully completed the protocol (72 in the control group and 72 in the TA group). All included women, apart from the one who did not meet the inclusion criteria, were included in the ITT analysis (Figure 2).

Anthropomorphic, obstetric and anaesthetic characteristics (Table 1), as well as PPH management (Table 2), were not significantly different between the two groups. There were no site-specific differences in any variables presented between the centres.

**Table 1:** Maternal and obstetric characteristics^a^

Group	TA	Control	*P *value
Number of patients	72	72	
Mean age, yr (± SD)	29 (4)	28 (5)	0.55
Mean weight, kg (± SD)	67 (16)	65 (12)	0.54
Mean height, cm (± SD)	164 (5)	165 (6)	0.18
Parity: primiparae, *n *(%)	46 (64)	50 (69)	0.06
Mean gestational age, weeks (± SD)	39.5 (2)	39.5 (1.8)	0.97
Twin pregnancies, *n *(%)	4 (6)	3 (4)	0.6
Abnormal placental insertion, *n *(%)	2 (3)	3 (4)	0.8
Oxytocin for labour induction, *n *(%)	9 (12)	12 (17)	0.88
Mean labour duration, hours (± SD)	6 (3)	6 (3)	0.82
Epidural analgesia, *n *(%)	59 (82)	61 (84)	0.45
Instrumental delivery, *n *(%)	7 (9)	10 (14)	0.85
Oxytocin at delivery, *n *(%)	30 (42)	31 (42)	0.89
Mean newborn weight, g (± SD)	3,475 (610)	3,489 (526)	0.89
Mean minutes between delivery and inclusion (± SD)	56 (49)	44 (41)	0.39
Atony-related PPH, *n *(%)	54 (75)	50 (69)	0.41

^a^SD, standard deviation; PPH, postpartum haemorrhage; TA, tranexamic acid. α risk: *P *< 0.05 using χ^2 ^test or the Fisher's exact test for categorical variables. Student's *t*-test was used in cases of normal distribution; otherwise, the Mann-Whitney *U *test was used for numerical variables.

**Table 2:** PPH management^a^

Group	TA	Control	*P *value
Number of patients	72	72	
Mean crystalloid loading at T3, mL (± SD)	934 (575)	949 (712)	0.54
Mean colloid loading at T3, mL (± SD)	611 (500)	736 (459)	0.13
Mean total loading volume, mL, (± SD)	1,547 (722)	1,672 (787)	0.36
Prostaglandins for PPH, *n *(%)	36 (48)	34 (43)	0.74
Postpartum thromboprophylaxis, *n *(%)	16 (22)	14 (20)	0.8

^a^PPH, postpartum haemorrhage; TA, tranexamic acid; T3, 2 hours after inclusion. α risk: *P *< 0.05 using the χ^2 ^test or Fisher's exact test for categorical variables. Student's *t*-test was used in cases of normal distribution, and otherwise the Mann-Whitney *U *test was used for numerical variables.

**Figure 3:**
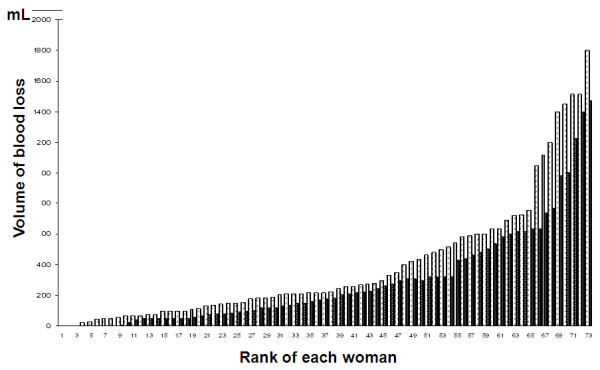
**Bar graph illustrating blood loss between T1 and T4 (from the smallest to the largest) for each woman in the two groups**. Black bars = TA group, white bars = control group. The *y*-axis represents the volume of blood loss (in millilitres) between T1 and T4. The *x*-axis values are the rank of each woman according to the amount of blood loss.

At the time of patients' inclusion (T1), blood loss did not differ between the two groups (median values of 1,000 mL (first to third quartiles, 840 to 1,110) in the TA group and 950 mL (first to third quartiles, 800 to 1,100 in the control group) (*P *= 0.96). The volume of each patient's blood loss in the two groups is shown Figure 3. The blood loss between T1 and T4 was significantly lower in the TA group (median, 170 mL (first to third quartiles, 58 to 323)) than in the control group (median, 221 mL (first to third quartiles, 110 to 543) (*P *= 0.041).

**Figure 4:**
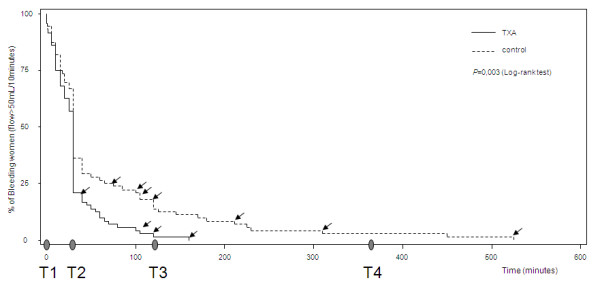
**Graph showing time from enrolment until PPH cessation in the two groups**. Solid line = TA group, dashed line = control group. *P *= 0.003 using the Kaplan-Meier logrank test. Time points of the study (T2 = T1 + 30 minutes, T3 = T1 + 2 hours, T4 = T1 + 6 hours) are indicated on the *x*-axis. The time of each invasive procedure is indicated by an arrow.

The duration of bleeding was lower in the TA group than in the control group (*P *= 0.004; logrank test) (Figure 4). Bleeding was stopped by T2 in 63% of women in the TA group and in 46% of women in the control group (*P *= 0.034).

**Figure 5:**
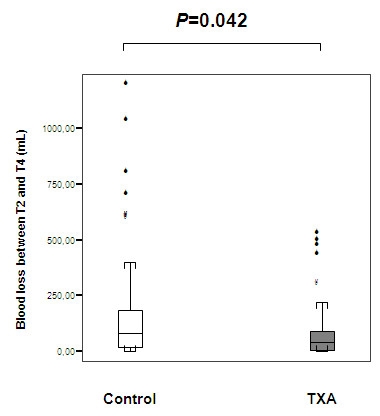
**Graph illustrating blood loss between T2 and T4 between the two groups**. *P *= 0.04 using the Mann-Whitney *U *test after applying the Bonferroni correction.

Because the time course of bleeding appeared to differ significantly after T2, we analyzed the volume of blood loss from T2 to T4. Between T2 and T4, blood loss was 49% lower in the TA group (median, 39 mL (first to third quartile, 2 to 101)) than in the control group (median, 77 mL (first to third quartile, 15 to 185)) (*P *= 0.03 after Bonferroni correction) (Figure 5).

The time at which invasive procedures were performed is shown in Figure 4. Haemostatic embolisation was performed in five women in the TA group and in five women in the control group (*P *= 0.94) after a median PPH duration of 110 minutes (range, 30 to 155 minutes) in the TA group and 140 minutes (range, 75 to 315 minutes) in the control group. Hysterectomy or surgical uterine artery ligature was performed in two women in the control group at 315 minutes and 525 minutes, respectively, and in none in the TA group.

PPH-related outcome ITT and per protocol analysis are both presented in Table 3. There was a trend toward a decrease in incidence of severe PPH in ITT analysis that was significant in per protocol analysis. The incidence of decrease in haemoglobin concentration of more than 4 g/dL, as well as the number of PRBCs transfused before day 42, was significantly lower in the TA group than in the control group in both analyses.

**Table 3:** Assessment of PPH-related outcome^a^

Group	TA	Control	*P *value
Number of patients			
ITT	77	74	
Per protocol	72	72	
Evolution to severe PPH, *n *(%)			
ITT	27 (35)	37 (50)	0.07
Per protocol	23 (32)	36 (50)	0.028
Persistent bleeding at T2, *n *(%)			
ITT	28 (36)	40 (54)	0.03
Per protocol	26 (36)	38 (53)	0.044
Haemoglobin drop >4 g/dL, *n *(%)			
ITT	19 (25)	32 (43)	0.02
Per protocol	15 (21)	34 (47)	< 0.001
PRBC transfusion before T4, *n *(%)			
ITT	10 (13)	13 (18)	0.17
Per protocol	7 (10)	12 (17)	0.65
PRBC units administered before T4, *n*			
ITT	32	62	0.26
Per protocol	18	38	0.4
PRBC transfusion total through day 42, *n *(%)			
ITT	13 (17)	20 (27)	0.33
Per protocol	9 (13)	20 (28)	0.16
PRBC units administered total through day 42, *n*			
ITT	28	62	< 0.001
Per protocol	24	62	< 0.001
Arterial embolisation, *n *(%)			
ITT	5 (6.8)	5.1 (6.1)	1
Per protocol	4 (6.0)	5 (7.0)	0.73
Surgical arterial ligature or hysterectomy, *n *(%)			
ITT	0	2 (2.7)	0.24
Per protocol	0	2 (3.0)	0.5
Late postpartum curettage (after day 7), *n *(%)			
ITT	1 (1.3)	2 (2.7)	1
Per protocol	1 (1.4)	2 (2.8)	1
Any vasopressor, *n *(%)			
ITT	4 (5.2)	4 (5.4)	1
Per protocol	3 (4.2)	4 (5.5)	1
Intensive care unit stay, *n *(%)			
ITT	3 (3.9)	5 (6.7)	1
Per protocol	3 (4.2)	5 (7.0)	1
Mild dyspnea, *n *(%)			
ITT	0 (0)	1 (1.3)	1
Per protocol	0 (0)	1 (1.4)	1
Multiple organ failure, *n *(%)			
ITT	0 (0)	0 (0)	-
Per protocol	0 (0)	0 (0)	-

^a^PPH, postpartum haemorrhage; TA, tranexamic acid; ITT, intention to treat; PRBC, packed red blood cell. α risk: *P *< 0.05 using the χ^2 ^test or Fisher's exact test for categorical variables. Student's *t*-test was used in cases of normal distribution, and otherwise the Mann-Whitney *U *test was used for numerical variables.

Overall, PPH reached the criteria for severity in 27 women in the TA group and in 37 women in the control group (*P *= 0.028). The subgroup of women who experienced severe PPH was not different from those who did not experience severe PPH with respect to anthropomorphic and obstetric characteristics, except for baseline volume of bleeding, which was significantly higher at T1 (median, 1,000 mL (first to third quartile, 870 to 1,200) in women with severe PPH vs. 900 mL (first to third quartile, 800 to 1,100) in women without severe PPH (*P *= 0.038). In those women with severe PPH, (1) haemorrhage duration was shorter in the TA group than in the control group (median 30 minutes (first to third quartile, 15 to 40 minutes) vs. median 30 minutes (first to third quartile, 20 to 93 minutes) (*P *= 0.001), and (2) in the PPH population, one woman in the TA group and seven women in the control group received procoagulant drugs (fibrinogen or FFP) for massive haemorrhage in accordance with practice guidelines and study design (*P *= 0.001).

Overall, PPH stopped after administration of uterotonic drugs and PRBC support and without any appropriate haemostatic drug (other than TA in the TA group ) in 57 women (79%) in the control group and in 67 women (93%) in the TA group (*P *= 0.016).

The only severe adverse manifestations were deep vein thrombosis at the site of the venous catheter, which occurred in two patients in the TA group and in one in the control group (*P *= 0.375) (Table 4). Urea, creatininemia, and diuresis at T4 did not differ between the two groups. Mild transient adverse manifestations (nausea, vomiting, dizziness and phosphenes) occurred more often in the TA group (*n *= 18) than in the control group (*n *= 4) (*P *= 0.03) (Table 4). No seizures and no maternal deaths occurred in either group.

**Table 4:** Side effects of treatment^a^

Group	TA	Control	*P *value
Number of patients			
ITT	77	74	
Per protocol	72	72	
Severe side effects			
Deep vein thrombosis, *n *(%)			
ITT	2 (3)	1 (1)	0.4
Per protocol	2 (3)	1 (1)	0.37
Renal failure, *n *(%)			
ITT	0 (0)	0 (0)	-
Per protocol	0 (0)	0 (0)	-
Mean T4 urea, g/L (± SD)			
ITT	0.17 (0.06)	0.2 (0.1)	0.9
Per protocol	0.1 (0.1)	0.2 (0.1)	0.9
Mean T4 creatininemia, mg/L (± SD)			
ITT	6.3 (1.8)	6.4 (1.7)	0.79
Per protocol	5.4 (2.8)	6.0 (2.3)	0.7
Mean T4 diuresis, mL (± SD)			
ITT	1,058 (1,010)	882 (480)	0.25
Per protocol	1,044 (933)	862 (575)	0.23
Seizures, *n *(%)			
ITT	0 (0)	0 (0)	-
Per protocol	0 (0)	0 (0)	-
Maternal death, *n *(%)			
ITT	0 (0)	0 (0)	-
Per protocol	0 (0)	0 (0)	-
Nonsevere side effects			
Nausea/vomiting, *n *(%)			
ITT	12 (15)	1 (2)	0.002
Per protocol	11 (15)	1 (2)	0.002
Phosphenes, *n *(%)			
ITT	9 (12)	2 (3)	0.02
Per protocol	8 (11)	2 (3)	0.02
Dizziness, *n *(%)			
ITT	4 (5)	3 (4)	0.28
Per protocol	4 (6)	3 (4)	0.28
Total nonsevere adverse effects, *n *(%)			
ITT	18 (23)	4 (6)	0.03
Per protocol	17 (24)	4 (6)	0.03

^a^TA, tranexamic acid; ITT, intention to treat. α risk: *P *< 0.05 using the χ^2 ^test or Fisher's exact test for categorical variables. Student's *t*-test was used in cases of normal distribution, and otherwise the Mann-Whitney *U *test was used for numerical variables.

## Discussion

This study demonstrates for the first time that TA administered to women with overt PPH decreases blood loss, bleeding duration and maternal morbidity with only minor, transient side effects. In addition, TA-treated women received fewer additional procoagulant treatments, such as FFP, platelets and fibrinogen.

### PPH definition and blood loss measurement

PPH is usually defined as blood loss >500 mL after vaginal haemorrhage [13,15], but it was defined as ≥400 mL blood loss in the studies by Gay *et al. *[12] and Yang *et al. *[14]. In the present study, we chose to include women who had blood loss >800 mL to select women with a high risk of severe PPH, thereby strengthening our results. Another important strength of this study is the careful and homogeneous measurement of blood loss in each participant using specially designed under-buttocks drapes with a graduated collection pouch that accurately evaluates small volumes. This measurement was completed by weighing the pouch and compresses. We also established a definition of bleeding flow to align the criteria for obstetric and intensive care decisions at each step of the procedure.

### Choice of the antifibrinolytic agent and doses

TA was chosen because it has been demonstrated to be a potent antifibrinolytic agent in elective surgical patients and because it is the most often used antifibrinolytic agent worldwide. TA has the additional advantage of being inexpensive and easy to stock and handle [10]. It remains the only antifibrinolytic agent available in France at present.

Given the lack of previous studies on PPH, we chose a fixed-dose regimen, which, given the weight of the participants, was, on average, a 60 mg/kg loading dose followed by a 16 mg/kg/hour infusion.

The high dose of 4 g + 6 g (60 mg/kg as a loading dose followed by a 16 mg/kg/hour infusion) TA was chosen in our study as the best clinically effective dose used to reduce haemorrhage in high-risk cardiac surgery patients [17,18,20,21]. At the beginning of the study, these were the only data available on active doses in reducing haemorrhage. This high dose has been used successfully since 2004 in high-risk cardiac surgery [21].

The purpose of this study was to investigate the potential for reducing bleeding by administering TA in women with active PPH. The studied population was selected on the basis of active haemorrhage of more than 800 mL when its clinical course might be life-threatening. The unusual 800-mL threshold for the definition of PPH, rather than 500 mL, was selected for active PPH. This selection of patients required a specific procedure for measurement and verification of blood loss at each time point.

Since then, the BART study in 2008 [22] and the CRASH-2 study in 2010 [11] have used lower doses of TA (30 mg/kg + 16 mg/kg/hour and 1 g + 1 g, respectively). In the BART study, patients were selected for their potential for high blood loss estimated on the basis of their risk of requiring surgery. In the CRASH-2 study, the patients were selected as patients "experiencing or considered to be at risk of significant haemorrhage" [11]. These studies' lower doses were designed to limit bleeding in a large and less selective population than that in our study.

### Clinical relevance of the results

The observed reduction in blood loss, although significant, was modest in terms of median values. Nonetheless, the time course of blood loss clearly suggests that TA prevented the onset of severe or intractable bleeding in some women. This suggestion was confirmed by the observation that the number of severe PPH cases was lower in the TA group than in the control group. The decrease in haemoglobin concentration and the need for blood transfusions were also reduced in the TA group. Finally, PPH stopped without administration of haemostatic drugs or invasive procedures in 93% of TA-treated women, but in only 80% of women in the control group. Therefore, we conclude that the mild effect of TA on median blood loss is clinically relevant and that TA may have prevented the need for procoagulant drugs or invasive procedures in up to 13% of women. An additional consequence of the decrease in maternal morbidity associated with TA is the potential to spare medical costs.

### Side effects

As in previous studies [10,11,22,23], no alteration of renal function was observed. Although this study was not powered to address safety issues, the only side effects we recorded were gastrointestinal and neurological manifestations as previously described [24,25], which were mild and reversible but were more frequent in the TA group than in the control group. We observed two cases of thrombosis in the TA group and one in the control group after complicated delivery and after TA treatment; however, the design of the study did not allow for a definite conclusion on the risk of thrombosis related to TA in this setting. That the high-dose regimen is responsible for the increased rate of side effects in the TA group remains possible.

### Potential limitations

First, the major weakness of this randomised, controlled study is its open-label, unblinded character. Therefore, the results are at risk of bias. This design was chosen to limit the budget, which was supported only by academic funding, and because of the restricted number of paramedics and medical teams available for PPH management, especially during on-call periods. However, centralized randomisation and strict data concealment were followed. Moreover, the anaesthesiologist performed randomisation and also immediately administrated (or not) the treatment. Although the study was not blinded, obstetricians and midwives were not aware of the treatment group, so the rest of the management, blood loss measurement and transfusion algorithm were conducted regardless of the group allocation. Finally, statistical analyses were performed on an ITT basis.

A second limitation is that the design of this study was not powered to show decreases in maternal death or number of invasive procedures, which are the ultimate goals of maternity treatment. Nevertheless, we observed a trend toward a decrease in the rate of PPH embolisation and surgical procedures. From this perspective, the study produced encouraging data that support the need for further work, such as the recently launched WOMAN trial [26], to assess the most important issues related to the reduction of maternal mortality.

Third, the TA-related risk of thrombosis evaluation could not be evaluated in this study, as deep vein thrombosis was only diagnosed clinically and confirmed by Doppler ultrasound. Twenty-two of the patients in each group were treated with thromboprophylaxis, as recommended for the PPH inflammatory syndrome. The power of the study does not allow for a definite conclusion regarding the risk of thrombosis related to TA in this setting.

Fourth, our study was performed in tertiary care and secondary care women's hospitals in a high-income country, which allowed for optimal obstetrical management. Whether these results can be reproduced in a suboptimal environment remains to be demonstrated. This factor is important to consider, since TA has the clear advantage of being an inexpensive, stable, off-the-shelf, easy-to-use drug, even in low-income countries.

## Conclusions

This study is the first to demonstrate that TA can reduce blood loss and maternal morbidity in ongoing PPH. Adverse effects were only mild and transient, even at the relatively high doses used, but the study was not powered to address safety issues. These encouraging data strongly support the need for a large, international, double-blind study to investigate the potential of TA to reduce maternal morbidity worldwide.

## Key messages

• We conducted a randomised, controlled study of 144 patients with the purpose of appreciating the effect of a high dose of intravenous tranexamic acid on strictly measured PPH volume.

• This study was conducted in eight French obstetrics units in accordance with French PPH treatment guidelines and was funded and monitored by public health academic support.

• We observed a significant reduction of blood loss, evolution to severe PPH, haemoglobin drop >4 g/dL, and a reduced number of PRBCs transfused before day 42.

• This study represents the first demonstration that antifibrinolytic treatment can decrease blood loss and maternal morbidity in women with PPH, which is a leading cause of maternal death.

• This study supports the need for a large international study to investigate the potential of TA, a simple and inexpensive treatment, to reduce maternal morbidity worldwide.

## Abbreviations

CRASH: Clinical Randomisation of an Antifibrinolytic in Significant Haemorrhage; CNIL: Commission Informatique et Liberté; FFP: fresh frozen plasma; ITT: intention to treat; PRBCs: packed red blood cells; PPH: postpartum haemorrhage; TA: tranexamic acid.

## Competing interests

The authors declare that they have no competing interests.

## Authors' contributions

ASDB contributed to the study's conception and design, as well as to acquisition of data, data management, analysis and interpretation of data, and drafting and revising the final manuscript submitted for publication. BJ and AD contributed to the study's conception and design as well as to acquisition of data, data management, analysis and interpretation of data, and drafting and revising the manuscript. FB, CH, HKM, LM, NT, SF, FLG and SDM contributed to the study's conception and design, the acquisition of data, and drafting and revising the manuscript. BV contributed to drafting and revising the manuscript. The EXADELI study group contributed to participant enrolment and acquisition of data. SS contributed to the study's conception and design, the analysis and interpretation of data, and drafting and revising the final version of the manuscript submitted for publication.
